# Investigation of an Elevational Gradient Reveals Strong Differences Between Bacterial and Eukaryotic Communities Coinhabiting *Nepenthes* Phytotelmata

**DOI:** 10.1007/s00248-020-01503-y

**Published:** 2020-04-14

**Authors:** Kadeem J. Gilbert, Leonora S. Bittleston, Mark Arcebal K. Naive, Anthony E. Kiszewski, Perry Archival C. Buenavente, David J. Lohman, Naomi E. Pierce

**Affiliations:** 1 Department of Organismic and Evolutionary Biology, Harvard University, 26 Oxford St., Cambridge, MA 02138 USA; 2 Department of Entomology, The Pennsylvania State University, 501 Agricultural Sciences and Industries Building, University Park, PA 16802 USA; 3 Department of Civil and Environmental Engineering, Massachusetts Institute of Technology, 77 Massachusetts Avenue, Room 1-290, Cambridge, MA 02139 USA; 4 Department of Biological Sciences, Boise State University, 1910 W University Dr, Boise, ID 83725 USA; 5 Department of Biological Sciences, College of Science and Mathematics, Mindanao State University-Iligan Institute of Technology, Andres Bonifacio Ave, 9200 Iligan, Lanao del Norte Philippines; 6 Department of Natural and Applied Sciences, Bentley University, 175 Forest Street, Waltham, MA 02452 USA; 7 Entomology Section, National Museum of Natural History, Manila, Philippines; 8 Biology Department, City College of New York, City University of New York, New York, NY USA; 9 Ph.D. Program in Biology, Graduate Center, City University of New York, New York, NY USA

**Keywords:** Arthropods, Bacteria, Elevational diversity gradients, Fungi, Phytotelmata, Protists

## Abstract

Elevation is an important determinant of ecological community composition. It integrates several abiotic features and leads to strong, repeatable patterns of community structure, including changes in the abundance and richness of numerous taxa. However, the influence of elevational gradients on microbes is understudied relative to plants and animals. To compare the influence of elevation on multiple taxa simultaneously, we sampled phytotelm communities within a tropical pitcher plant (*Nepenthes mindanaoensis*) along a gradient from 400 to 1200 m a.s.l. We use a combination of metabarcoding and physical counts to assess diversity and richness of bacteria, micro-eukaryotes, and arthropods, and compare the effect of elevation on community structure to that of regulation by a number of plant factors. Patterns of community structure differed between bacteria and eukaryotes, despite their living together in the same aquatic microhabitats. Elevation influences community composition of eukaryotes to a significantly greater degree than it does bacteria. When examining pitcher characteristics, pitcher dimorphism has an effect on eukaryotes but not bacteria, while variation in pH levels strongly influences both taxa. Consistent with previous ecological studies, arthropod abundance in phytotelmata decreases with elevation, but some patterns of abundance differ between living inquilines and prey.

**Electronic supplementary material:**

The online version of this article (10.1007/s00248-020-01503-y) contains supplementary material, which is available to authorized users.

## Introduction

Mountains, especially tropical mountains, can exhibit differences in climatic variables across their ranges comparable to the climatic changes that occur around the globe from equator to poles [1–4]. Because mountains create spatially compact environmental gradients, they are useful for exploring broad patterns across taxa. Decades of research has uncovered similar patterns across diverse taxa: whether monotonic or hump-shaped, plants, mammals, birds, amphibians, reptiles, insects, and other invertebrates all generally exhibit decreasing diversity and abundance with increasing elevation [5]. While studies of elevational gradients can be traced back to the time of Linnaeus, bacteria and other microbes have been the focus of such studies only recently—largely within the past decade [6]. The extent to which microbes follow the same macroecological trends established in plants and animals (macro-organisms or “macrobes”) remains partially unresolved. One major line of thought on microbial macroecology is the Baas-Becking hypothesis: “everything is everywhere, and the environment selects”, suggesting that bacteria are not dispersal-limited and thus not subject to the broad-scale spatial structuring seen in macrobes [7–12]. However, this view is controversial [13–17], and may not apply to eukaryotic microbes [18, 19].

Microbes have not been as extensively studied in the context of elevational gradients, but the past decade has seen an increasing number of studies examining elevational patterns of bacteria in soil [6, 8–12, 20–22] and to a lesser extent, aquatic habitats [23–26]. Similar studies have also been conducted for fungi [27–35] and protists [18, 36–39]. Of these microbial elevational gradient studies, only a handful have compared diversity patterns of syntopic microbes and macrobes coinhabiting the same microhabitats. The results of these studies have also been inconsistent, with some finding that microbes and macrobes follow concordant patterns of richness (generally decreasing richness with increasing elevation) [6, 22], and others finding that microbes and macrobes exhibit differing patterns [21, 25, 40]. Other, more general analyses have found that bacteria and micro-eukaryotes respond differently to environmental factors within the same habitats, with eukaryotes having stronger dispersal limitation [41–45]. It may thus be necessary to distinguish between bacteria and eukaryotes when comparing macrobes to microbes.

A multi-taxon comparative approach can contribute to improved understanding of elevational macroecology. Including organisms with diverse physiologies spanning multiple trophic levels helps improve the generalizability of observed patterns [46]. To achieve this task, our study utilizes communities in phytotelmata, which are small, specialized aquatic ecosystems contained within plant tissues such as tree holes or pitcher plants [47]. These systems have unique advantages for comparative multi-taxon studies: though small, they often encompass a diverse set of taxa, which can include bacteria, archaea, eukaryotic microbes, invertebrates, and vertebrates (the term “inquilines” has been used to refer to phytotelm inhabitants, especially invertebrates) [47]. Thus, they serve as convenient, replicated communities with distinct boundaries in which multiple taxa experience a similar environment and can be compared simultaneously using a metabarcoding approach [48]. Specifically, we studied tropical pitcher plants (genus *Nepenthes*), which are carnivorous plants with modified leaves or “pitchers.” These pitchers act as pitfall traps, in which a secreted fluid pool digests captured insects, while also serving as a phytotelm habitat [49, 50]. Trait variation in pitchers, both morphological and physiological, can also influence community composition. Morphological features include pitcher dimorphism (an individual plant produces two distinct pitcher morphs, “lower” and “upper” pitchers from the terrestrial rosette and arboreal climbing phases, respectively) and variation in red pigmentation [51, 52]. Coloration may affect visual signaling to arthropods [52, 53]. Pitcher dimorphism may involve differences in visual and olfactory signaling modalities in addition to the terrestrial/arboreal microhabitat distinction [54, 55]; two studies found between-morph differences in inquiline insect community composition [56, 57]. One key physiological feature of pitchers is their active regulation of fluid pH levels [58], which can vary significantly within or between species [59, 60]. *Nepenthes* phytotelm communities typically include bacteria, fungi, algae, protozoans, mites, and aquatic insect larvae [47, 49, 59], and, occasionally, anuran tadpoles [61–63] and crabs [64]. Previous metabarcoding studies show that their communities are specialized and distinct from the surrounding environment [65], which can be attributed to the specific conditions within the plants such as the acidic pH levels of the fluids [65, 66].

In this study, we examine phytotelm communities of *Nepenthes mindanaoensis* along a 400–1200 m a.s.l. elevational gradient on Mt. Hamiguitan, Mindanao, Philippines. We collected the entire fluid contents and used a DNA metabarcoding approach to sequence 16S and 18S rRNA genes to capture the community-level diversity of bacteria and eukaryotes, respectively. The sequence-based approach captures both micro- and macro-eukaryotes (i.e., metazoans). We also identified and counted physical specimens of arthropods in the pitcher, both the inquilines (generally aquatic insect larvae that complete their development living and feeding inside the pitcher fluids), as well as the partially digested prey remains. Our primary goal was to determine whether pitcher phytotelm communities are structured by elevation, or whether plant-regulated factors such as pH and morphology have a greater effect that overrides the effect of elevation (which might be largely generated by external climatic factors such as temperature and precipitation). We were also interested in whether the relative effects of external gradients differed among taxa. We did not attempt to establish the effect of specific climatic factors, and unfortunately elevation and geographic distance covaried in our transect, so it was not statistically possible to disentangle the effects of these separate external (i.e., non-plant-regulated) factors on pitcher biota. However, we still gain insight into the relative influence of plant-regulated versus external factors. Whether the influence of the external gradient is due to climate or distance (or a combination of both), determining its relative effect on different taxa provides information of interest, since the comparative effect of geographic distance on microbes and macrobes is also an open question [42, 67].

## Methods

### Site

Mount Hamiguitan (N 06° 43′ 1.81″, E 126° 10′ 24.35″) is located in the southernmost peninsula of eastern Mindanao, the southernmost major island of the Philippines. Much like the neighboring Indo-Australian Archipelago, the Philippines is a hotbed of biodiversity. Because the majority of its islands were never connected to mainland Southeast Asia, the Philippines boasts impressive levels of endemism: an estimated 45% of vertebrate species, 50% of plant species, and 70% of insect species are endemic [68]. Further, many species are endemic to single islands or even single peaks. Five *Nepenthes* species are endemic to Mt. Hamiguitan [69, 70]*.* This site-level endemism is one reason for Mt. Hamiguitan Range Wildlife Sanctuary’s designation as an UNESCO World Heritage Site. The site ranges from 75 to 1637 m a.s.l. and has several habitat types, including dipterocarp forest, montane forest, moss forest, and pygmy moss forest at the highest elevation.

Following the general pattern observed on tropical mountains, there is a pronounced gradient in both temperature and precipitation. We determined the site’s climatic properties using data from WorldClim [71], analyzed using the “st” package in R [72], with a resolution of 5 min for max temperature and 2.5 min for all other variables. In any given month, the mean temperature for the lowest elevation region of Mt. Hamiguitan is 26–27 °C compared to 21–22 °C at the highest elevation, while the monthly minimum temperature ranges from 21 to 22 °C at the lowest elevation and 16–18 °C at the highest elevation. For maximum temperature, the range is 30–30.5 °C at low elevation and 27.5–29 °C at high elevation. Precipitation patterns are more variable throughout the year than temperature patterns, but during the months of June to September (monsoon season), conditions tend to be wetter at low elevation: 18–36 mm at the lowest elevation compared to 17–24 mm at the highest elevation (Supplementary Table 1).

### Sample Collection

**Fig. 1:**
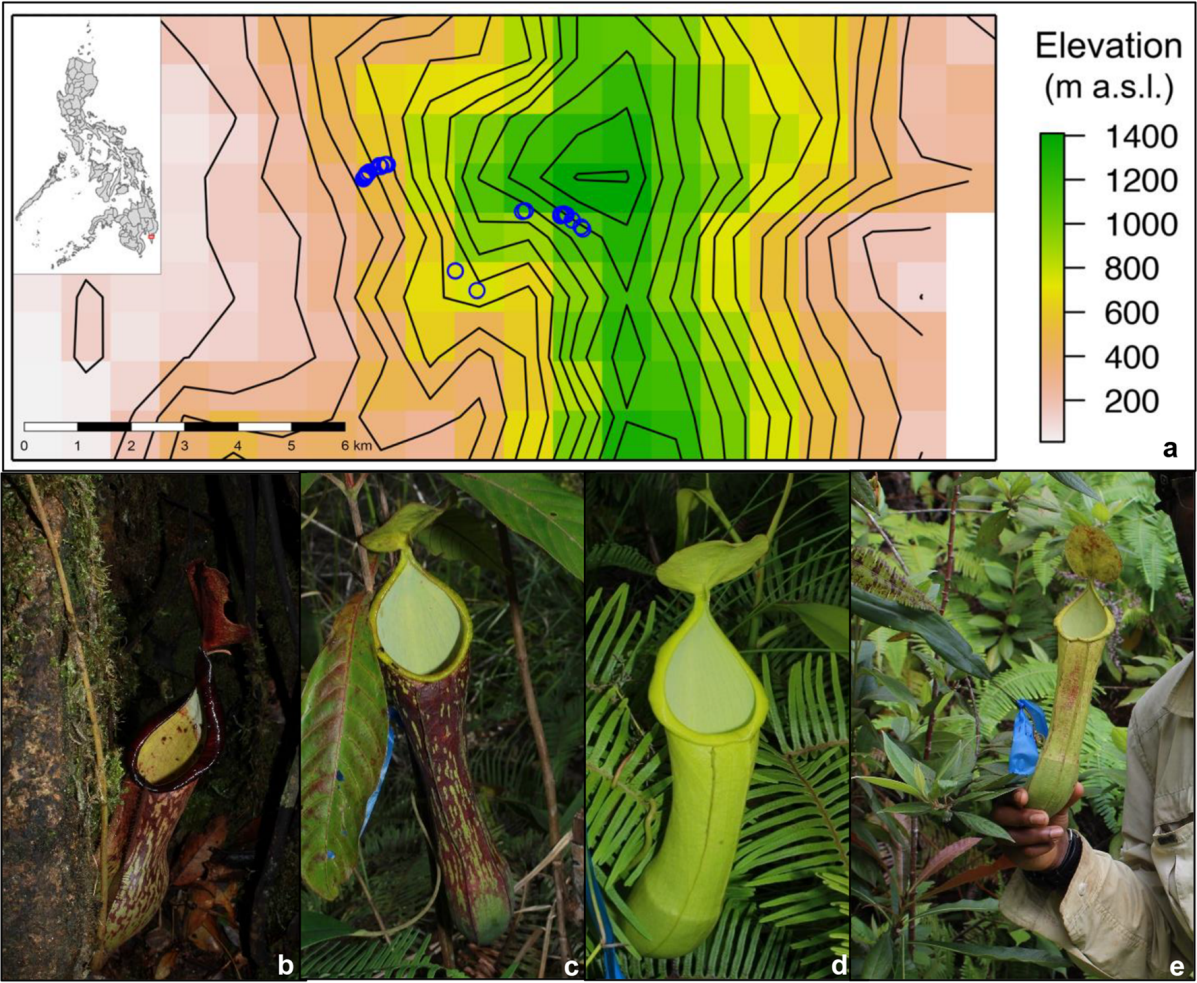
**a** Sampling area for the study, blue circles indicate the locations of the sampled pitchers on Mount Hamiguitan. Location of the area within the Philippines is indicated by a red box in the inset. **b**–**e** Representative photographs of *N*. *mindanaoensis* pitchers sampled in this study: lower pitcher (**b**) and upper pitchers (**c**–**e**). Photos: MAKN

Sampling was conducted 14–17 July 2016. We worked along a transect from ~ 400 to ~ 1200 m a.s.l. (a Euclidian distance of ~ 4.2 km, Fig. 1). *Nepenthes mindanaoensis* individuals were abundant throughout this transect, allowing for systematic sampling; however, we sampled fewer pitchers from ~ 600 to 800 m a.s.l. due to more challenging terrain. We collected the contents of 33 *N*. *mindanaoensis* pitchers (Table 1), selecting only a single healthy mature pitcher per individual plant. We poured the entire fluid contents from each pitcher directly into a single, sterile 50 mL Falcon tube, sealing the capped tubes with parafilm to safeguard against spillage or contamination prior to the addition of preservative. The tubes were kept secure and out of the sun within a backpack during in-field transit. We added 1 mL of cetyl trimethylammonium bromide (CTAB) buffer for every 1 mL of pitcher fluid within 24 h of collection as a preservative (this was done indoors at a field station) [65]. Prior to the addition of CTAB, we recorded the volume of each sample and removed a small amount of liquid to measure pH with ColorPhast pH strips (Merck KGaA Darmstadt, Germany). We measured the length (distance from the base of the pitcher to the insertion of its lid) and width (the diameter of the widest section of the pitcher) of each pitcher in situ using digital calipers. We also recorded pitcher morph (upper or lower) and color (primarily green or red-pigmented) of each sample. We estimated “canopy openness” for each pitcher by photographing the sky above from pitcher’s-eye-view using a point-and-shoot digital camera (Canon PowerShot ELPH 170IS, which has a 4.5–54 mm zoom lens with 12× digital zoom) and calculating the area not covered by vegetation using ImageJ [73]; this metric does not only include leaf area of canopy trees but also all herbaceous layers shading that pitcher. We obtained GPS coordinates and elevation of each sampled pitcher using a Garmin eTrex handheld GPS unit. We determined Euclidian distance between plants using the distance tool in Google Earth Pro.

**Table 1:** List of all *Nepenthes mindanaoensis* pitcher fluid samples collected, with data on pitcher morph, elevation, and Euclidean distance from start of transect

Pitcher ID	Morph	Elevation (m a.s.l)	Euclidean distance from start (m)
MIN001	Upper	446	0
MIN002	Upper	447	6.89
MIN003	Upper	451	28.21
MIN004	Upper	446	28.21
MIN005	Upper	458	59.39
MIN006	Lower	459	72.61
MIN007	Lower	468	88.05
MIN009	Upper	465	102.25
MIN008	Lower	464	106.06
MIN010	Upper	464	108.02
MIN011	Upper	471	110.66
MIN012	Upper	475	154.95
MIN013	Upper	506	368.83
MIN014	Upper	514	385.3
MIN015	Upper	525	455.97
MIN016	Lower	523	462.43
MIN017	Upper	533	476.6
MIN018	Lower	533	490.63
MIN019	Upper	533	499.06
MIN020	Upper	539	511.12
MIN022	Upper	791	2249.2
MIN021	Upper	791	2253.75
MIN035	Upper	837	2755.37
MIN023	Lower	1003	3082.09
MIN024	Lower	1008	3107.74
MIN026	Lower	1042	3763.74
MIN025	Lower	1054	3776.82
MIN027	Lower	1087	3822.08
MIN028	Lower	1120	3870.16
MIN029	Lower	1112	3884.43
MIN030	Upper	1111	3884.43
MIN034	Lower	1202	4214.95
MIN033	Lower	1200	4248.15

### Arthropod Identification and Analysis

After removing fluid for DNA extraction, we filtered arthropod bodies and debris from fluid samples using fine gauze (< 0.5 mm pore size) and separated taxa under a dissecting microscope. We created high-resolution digital images using a digital camera mounted on a dissecting microscope together with the AutoMontage photo compositing system, then stored the specimens in 100% ethanol. We counted total arthropod numbers from each pitcher. For arthropods classed as insect prey, numbers were based on a combination of head capsule counts and wing counts; in the case of wings, we counted two morphologically similar wings as one individual. Arthropod counts were categorized as culicid (mosquito) larvae, ceratopogonid (midge) larvae, brachyceran (a dipteran suborder) larvae, mites (Acari), ants (Formicidae), and other insect prey. We used field guides to key ants to species where possible [74], and culicids to genus [75]. Order-level designation of insect prey was based on wing venation patterns or the appearance of head capsules. For further statistical analysis, both arthropod abundance (number of individual arthropods within a taxonomic category) and richness (number of taxa within a category, morphospecies in the case of ants and culicids, or orders in the case of non-ant insect prey) were based on physical counts, rather than 18S sequences.

### Extraction and Sequencing

We used metabarcoding to sequence the 16S and 18S ribosomal RNA genes in the fluid to represent the entire prokaryotic and eukaryotic communities in the pitcher fluid. DNA was extracted using a bead-beating and phenol-chloroflorm extraction method after concentrating the cells with a centrifuge [76]. Negative controls were included for each set of extractions, and no measurable DNA was recovered from them. Amplicons were generated and sequenced at the Environmental Sample Preparation and Sequencing Facility (ESPSF) at Argonne National Laboratory. We targeted the V4 region of the 16S rRNA gene using primers 515F-806R [77, 78] and the V9 region of the 18S genes using primers Euk1391f-EukBr [79, 80]. Sequences were assembled and assigned to operational taxonomic units (OTUs) using the QIIME pipeline and Harvard’s Odyssey computer cluster [65]. We used the Greengenes and SILVA databases for 16S and 18S sequences, respectively, for taxonomic classification of OTUs, with a cutoff of 97% sequence identity. In some cases, further taxonomic assignment was determined using NCBI BLAST. Neighbor-joining phylogenies were constructed for all bacterial (16S) and eukaryotic (18S) OTUs. 16S OTUs classified as chloroplast and mitochondrial sequences, and 18S OTUs classified as Embryophyta (land plant) sequences were removed from downstream analyses of community similarity to avoid inclusion of possible contaminants from host plant cells.

### Statistical Analysis

All analyses were conducted in R version 3.5.0. We first conducted a principal components analysis (PCA) of all recorded sample traits (elevation, Euclidian distance, fluid pH, canopy openness, fluid volume, pitcher length, pitcher width, pitcher morph, and pitcher color) in order to determine the axes of variation and assess correlation among traits. Based on this assessment, we determined that Euclidian distance (the shortest distance between two sampling points) was strongly, positively correlated with elevation (on the same PCA axis), and thus did not include Euclidian distance as a variable in our analyses. In order to further probe the effect of physical Euclidian distance on our data, we plotted the log of the Unifrac distance matrix for bacteria or eukaryotes against the log of Euclidian distance (in meters) to search for distance-decay patterns. One would expect to see stronger distance-decay patterns in more dispersal-limited taxa [81, 82]. In addition, we used the function “betadisper,” together with “permutest” to calculate and compare levels of 16S-/18S-based beta diversity (pitcher-to-pitcher turnover) between low (< 700 m a.s.l.) and high (> 700 m a.s.l.) elevation pitchers for bacteria and eukaryotes. Since high elevation pitchers were more widely distributed in the habitat than low elevation pitchers (maximum Euclidian distance of 1998.95 m vs. 511.25 m, respectively), we expected greater turnover at high elevations if inter-pitcher community similarity were strongly influenced by Euclidian distance. We chose to use pitcher length as the sole pitcher dimension because pitcher length is positively correlated with width, but unlike pitcher width, length is not correlated with fluid volume.

We analyzed 16S/18S-based community composition with the “vegan” R package, using the non-metric multidimensional scaling (NMDS) ordination method and the unweighted Unifrac distance metric. Samples were rarefied to 1311 (16S) and 1103 (18S) sequences. We assessed the significance of clustering by categorical variables (pitcher morph and color) using the “adonis” function in the “vegan” package [83], which performs a PERMANOVA test. For quantitative traits (elevation, pH, canopy openness, fluid volume, pitcher length), we performed Mantel tests. We calculated alpha diversity according to “effective number of species” [84], which can be interpreted as a direct measure of species richness, unlike standard diversity indices. We calculated effective number of species for 16S/18S data from Shannon Index values obtained using the function “diversity” in the “vegan” package.

To examine patterns of differential abundance of individual OTUs in relation to fluid properties, we performed the analysis of composition of microbiomes (ANCOM) [85], a test designed to examine taxon abundance while accounting for the fact that metagenomics studies yield relative abundance data as opposed to absolute abundance. For ANCOM tests, we used the full set of successfully extracted samples, only included OTUs with sequence counts above 100, and corrected for multiple testing (FDR) at a significance level of 0.05. Continuous environmental variables were transformed into categorical variables for ANCOM tests. We binned pH into three categories: low (≤ 3.0), mid (3.0–4.5), and high (≥ 5.0). Elevation was binned into three categories: low (400–600 m a.s.l.), mid (600–900 m a.s.l.), and high (> 900 m a.s.l.). Canopy openness was binned into three categories, which generally correspond to our preliminary qualitative assessments of canopy openness in the field: closed (~ 0–20% open), semi-open (~ 20–40% open), and open (~ 40–100% open).

In order to assess correlations of our measures with physical specimen-based arthropod counts, we conducted Poisson regressions using the “glm” function in the “lme4” package in R. We included all examined factors (elevation, pH, canopy openness, fluid volume, pitcher length, pitcher morph, and pitcher color) together into a single generalized linear model (Poisson regression), in order to account for correlations between factors. We conducted a separate regression each for the abundance of culicids, ceratopogonids, brachyceran larvae, mites, ants, and other insect prey and applied a Bonferroni correction in assessing significance for the family-wise set of six arthropod groups. The same method was used to assess correlations with richness for the family-wise set of three arthropod groups: ant morphospecies, culicid morphospecies, and non-ant insect prey orders.

## Results

### Factors Structuring Community Composition

Fluid pH is the only factor significantly structuring bacterial community composition (Mantel *r* = 0.64, *p* = 0.001; Table 2). While not significant at the Bonferroni-corrected alpha level of 0.007, bacteria appear to be somewhat structured by elevation as well, though to a lesser degree than pH (Mantel *r* = 0.22, *p* = 0.009). For eukaryotes, both elevation (Mantel *r* = 0.40, *p* = 0.002) and pH (Mantel *r* = 0.31, *p* = 0.004) have a significant effect on community composition, with elevation having a somewhat stronger effect. However, when Metazoa are excluded from the OTU table, elevation is the only factor with a significant effect (Mantel *r* = 0.35, *p* = 0.001). In this case, pH shows a possible effect though not significant (Mantel *r* = 0.31, *p* = 0.015; Table 2).

**Table 2:** Results of Mantel (^1^) or PERMANOVA (^2^) analyses of 16S- or 18S-based community composition for Bacteria, Eukaryotes, and Eukaryotes without Metazoa. The seven factors constitute separate tests on the same ordination for each of the respective three taxa, so the set of tests for each taxon is accordingly considered a family-wise set to account for multiple testing. “Coefficient” refers either to Mantel r or PERMANOVA *R*^2^ depending on the test

Factor	Bacteria		Eukaryotes		Eukaryotes without Metazoa	
	Coefficient	*p* value	Coefficient	p value	Coefficient	p value
Elevation^1^	0.220	0.009	0.400	0.002*	0.351	0.001*
pH^1^	0.640	0.001*	0.310	0.004*	0.300	0.015
Canopy openness^1^	0.020	0.350	0.070	0.130	0.020	0.399
Fluid volume^1^	0.060	0.280	0.060	0.280	0.023	0.397
Pitcher length^1^	0.002	0.468	− 0.050	0.700	− 0.095	0.803
Pitcher morph^2^	0.060	0.020	0.080	0.016	0.066	0.216
Pitcher color^2^	0.030	0.910	0.039	0.572	0.050	0.586

*Indicates significance at Bonferroni-corrected alpha level of 0.007

### OTU Taxonomic Composition

**Fig. 2:**
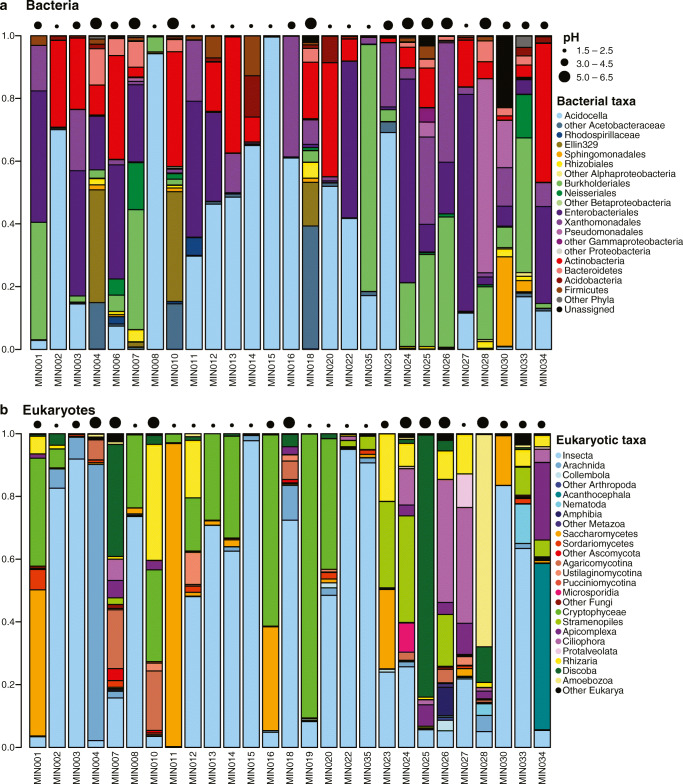
Stacked barplots showing relative abundances (sequence-based) of bacterial taxa (**a**) and eukaryotic taxa (**b**) as determined by 16S and 18S metabarcoding, respectively. Samples arranged by increasing elevation. The fluid pH of the samples is indicated by proportionally sized circles

We found a total of 2867 bacterial and 923 eukaryotic (including 405 metazoan) operational taxonomic units (OTUs), analogous to species. Proteobacteria—particularly Acetobacteraceae in Alphaproteobacteria, Burkholderiales in Betaproteobacteria, and Enterobacteriales in Gammaproteobacteria—dominate the bacterial composition (comprising on average 82% of all sequences); Actinobacteria, Bacteroidetes, and Firmicutes are also common across samples, with lower relative abundance (comprising on average 11%, 1.9%, and 1.2% of all sequences, respectively; Fig. 2). The eukaryotic communities consist of many taxa, including Metazoa, Alveolata, Stramenopiles (especially Chrysophyceae), Rhizaria, Cryptophyceae (especially *Goniomonas*), Discoba (primarily euglenids), Fungi, and Amoebozoa. Within Metazoa, Insecta is dominant (comprising on average 86% of all metazoan sequences), followed by Arachnida (specifically mites in Acari; 4.3% of sequences). Other arthropods and nematodes appear far less frequently and with lower relative abundance (Fig. 2). We observed frog eggs (from an unidentified rhacophorid) in one of the pitchers we sampled, and the 18S data was able to capture this (Fig. 2).

### Sequence-Based Alpha Diversity

Bacterial 16S-based alpha diversity (“effective number of species”, Jost [68]) does not significantly correlate with elevation (Table 3, glm, *t* value = 0.646, *p* = 0.526). The only factor that significantly correlates with bacterial alpha diversity is pH, with greater alpha diversity at higher pH (glm, *t* value = 3.401, *p* = 0.003). Eukaryotic 18S-based alpha diversity also does not significantly correlate with elevation (Table 3, glm, *t* value = − 0.283, *p* = 0.780). Neither does it significantly correlate with pH (glm, *t* value = 1.256, *p* = 0.225). The only factor that significantly correlates with eukaryotic alpha diversity is pitcher morph, with greater alpha diversity in lower pitchers (glm, *t* value = − 3.007, *p* = 0.007). When Metazoa are removed from the eukaryotic OTU table, no factor significantly correlates with alpha diversity (Table 3). Bacterial and eukaryotic alpha diversity do not clearly correlate with one another (linear model, *R*^2^ = 0.11, *p* = 0.09) unless metazoans are removed from the eukaryotic OTU table (linear model, *R*^2^ = 0.28, *p* = 0.006), in which case non-metazoan eukaryotic alpha diversity positively correlates with bacterial alpha diversity.

**Table 3:** Results of generalized linear model test of factors correlating with 16S- or 18S-based alpha diversity (“effective number of species”, Jost 2006). All factors included in one model to account for correlations between factors

Factor	Bacteria		Eukaryotes		Eukaryotes without Metazoa	
	*t* value	*p* value	*t* value	*p* value	*t* value	*p* value
Elevation	0.646	0.526	− 0.283	0.780	1.349	0.193
pH	3.401	0.003*	1.256	0.225	1.224	0.236
Canopy openness	0.238	0.815	− 1.182	0.252	0.273	0.788
Fluid volume	− 0.651	0.523	1.132	0.272	0.234	0.818
Pitcher length	0.543	0.594	1.492	0.152	0.848	0.407
Pitcher morph (uppers relative to lowers)	− 0.534	0.599	− 3.007	0.007*	− 1.63	0.120
Pitcher color (red relative to green)	− 0.795	0.437	− 1.373	0.186	− 1.351	0.192

*Significant at a Bonferroni-corrected alpha level of 0.0167

### Relative Abundance of Individual OTUs—ANCOM Tests

**Fig. 3:**
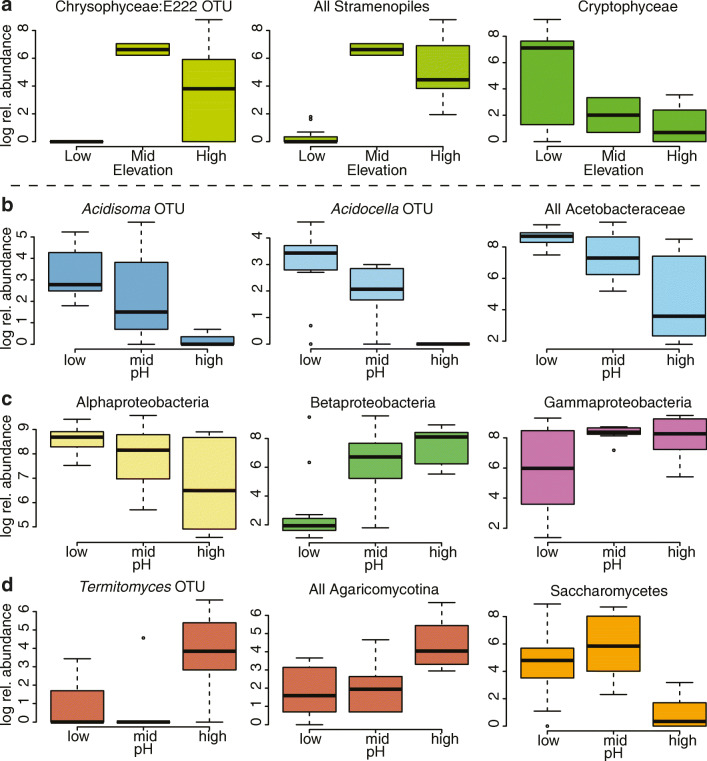
Boxplots showing results of ANCOM analysis by elevation (**a**) and pH (**b**–**d**). Elevation categories are low (400–600 m a.s.l.), mid (600–900 m a.s.l.), and high (> 900 m a.s.l.). We acknowledge that mid elevation is under-sampled (three pitchers) and is included here for illustrative purposes despite not being strictly statistically comparable to the other two categories. pH categories are low (≤ 3.0), mid (3.0–4.5), and high (≥ 5.0). In (**a**), we compared the one significantly differentially abundant OTU to the larger taxon to which it belongs and contrast it to an ecologically comparable taxon, Cryptophyceae. In (**b**), we compared the two significantly differentially abundant OTUs to the family to which they belong, as well as their class contrasted against two other Proteobacterial classes (**c**). In (**d**), we compared the one significantly differentially abundant OTU to the class to which they belong, and contrasted the pattern against another fungal class

The relative abundance of one bacterial OTU and one eukaryotic OTU varied significantly with elevation. The bacterial OTU is assigned to Acetobacteraceae (unclassified to genus); this OTU appears at low elevation, but not at mid or high. However, this trend is not representative of Acetobacteraceae in general, which show no differences in relative abundance across elevation categories (Kruskal-Wallis χ^2^ = 4.836, *p* = 0.089). The eukaryotic OTU is assigned to Chrysophyceae (Stramenopiles, “uncultured marine eukaryote E222”) and is present at mid and high elevation, but not at low. This reflects the trend in Stramenopiles in general, as all Stramenopile OTUs together are much less abundant at low elevation than mid and high elevation (Kruskal-Wallis χ^2^ = 20.84, *p* < 0.0001). While no Cryptophyceae OTUs were determined to be differentially abundant by elevation through the ANCOM test, Cryptophyceae in general (all classified as the genus *Goniomonas*) tend to be more abundant at low elevation, in contrast to the Chrysophyceae (Kruskal-Wallis χ^2^ = 6.561, *p* = 0.038; Fig. 3a).

The relative abundances of two bacterial OTUs and one eukaryotic OTU were significantly differentially abundant across pH categories. The bacterial OTUs include one classified in the genus *Acidisoma* and one in *Acidocella* (both Acetobacteraceae), which both tended to decrease with increasing pH. This trend reflects what can be seen for all Acetobacteraceae, with lower mean log relative abundance in the high pH category (Kruskal-Wallis χ^2^ = 11.663, *p* = 0.003, Fig. 3b). As Acetobacteraceae are the dominant representatives of Alphaproteobacteria, the trend also holds for Alphaproteobacteria in general (though not statistically significant, Kruskal-Wallis χ^2^ = 5.329, *p* = 0.07). This can be contrasted with Betaproteobacteria, which have higher mean log relative abundance at higher pH (Kruskal-Wallis χ^2^ = 10.327, *p* = 0.006), or Gammaproteobacteria which exhibit no pronounced trend with pH (Kruskal-Wallis χ^2^ = 3.516, *p* = 0.172; Fig. 3c).

The single eukaryotic OTU with significant differential abundance across pH categories was classified as belonging to *Termitomyces* (Agaricomycotina: Basidiomycota); the trend of higher relative abundance at high pH compared to low and mid pH categories is generalizable to Agaricomycotina (Kruskal-Wallis χ^2^ = 10.669, *p* = 0.005). This can be contrasted with Saccharomycetes (Ascomycota) which have lower relative abundance at high pH compared to low and mid pH categories (Kruskal-Wallis χ^2^ = 11.697, *p* = 0.003, Fig. 3d).

### Correlations Among Factors—Elevation and Euclidian Distance

**Fig. 4:**
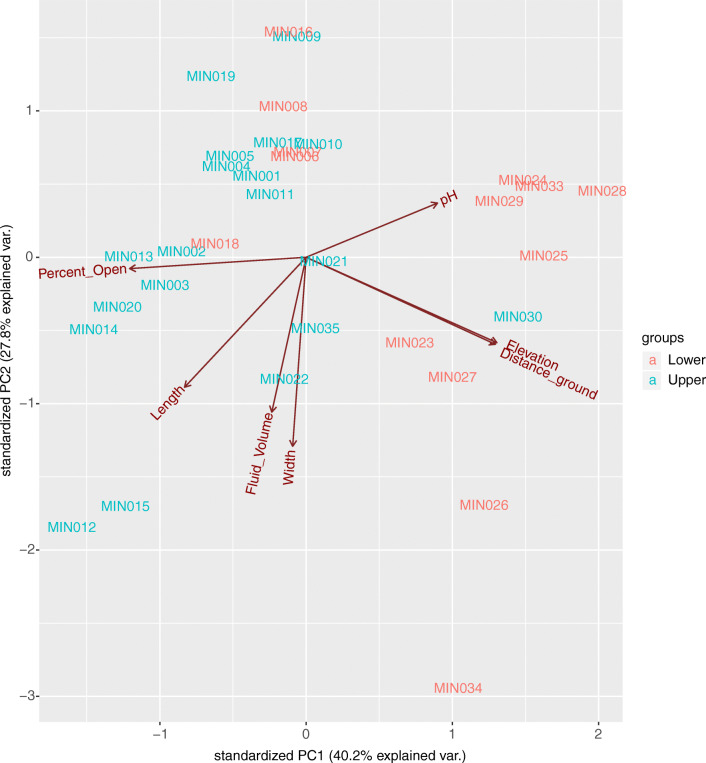
Principle components analysis (PCA) plot showing axes of variation for the metadata of all sampled pitchers: elevation (m a.s.l.), ground (Euclidian) distance (m), pH level, the amount of open sky above from the perspective of the pitcher (“Percent_Open,” i.e., canopy openness), the length and width of the pitcher (mm), and the volume of the fluid sample (mL). Points are labeled with the sample ID and colored by pitcher morph: “lower pitchers” in red and “upper pitchers” in blue

The seven continuously varying factors we recorded (elevation, Euclidian distance, canopy openness, pH, fluid volume, and pitcher length/width) differed considerably among our sampled pitchers (Fig. 4), with 68% of the total variation explained by PCA axes 1 and 2. As previously noted, elevation and Euclidian distance strongly covary. We did not find a distance-decay relationship for either bacteria or eukaryotes; rather, there is a slight positive correlation between community similarity and Euclidian distance for bacteria (*R*^2^ = 0.012, *p* = 0.038), eukaryotes (*R*^2^ = 0.077, *p* < 0.0001), and eukaryotes without Metazoa (*R*^2^ = 0.061, *p* < 0.0001). Bacteria show no significant difference in beta diversity (pitcher-to-pitcher turnover) between low elevation (average distance to median = 0.52) and high elevation (average distance to median = 0.51) in our analysis (permutation test for homogeneity of dispersions, *F* = 0.27, *p* = 0.59). Eukaryotes also show no significant difference in beta diversity between low (average distance to median = 0.462) and high (average distance to median = 0.457) elevation (permutation test for homogeneity of dispersions, *F* = 0.04, *p* = 0.85). Without Metazoa, eukaryotes still show no significant difference in beta diversity between low (average distance to median = 0.441) and high (average distance to median = 0.447) elevation (permutation test for homogeneity of dispersions, *F* = 0.04, *p* = 0.85). The lack of distance-decay patterns or change in beta diversity with elevation suggests that pitcher organisms are not dispersal limited across this transect.

### Community Composition, Richness, and Abundance of Arthropods—Physical Counts

We examined physical specimens of inquiline and prey arthropods to obtain count data. Physical count data differ from 18S sequence data. For instance, we did not obtain any OTUs assigned as ants, despite ants making up the majority of prey items we observed; this has previously occurred in *Nepenthes* metabarcoding studies [86] and may reflect primer biases against Formicidae. Unlike a previous study [86], OTU sequence counts in our study do not correlate well with physical specimen counts (linear regressions on log-transformed data, culicids: *R*^2^ = 0.04, *p* = 0.31; ceratopogonids: *R*^2^ = 0.005, *p* = 0.74; mites: *R*^2^ = 0.001, *p* = 0.88; non-ant insect prey: *R*^2^ = 0.006, *p* = 0.69). On the other hand, 18S data seem to accurately capture the presence of nematoceran (i.e., most inquiline dipterans) families and non-ant insect prey orders observed from physical specimens across fluid samples (Supplementary Fig. 1). Unless otherwise noted, all analyses of arthropod composition, richness, and abundance henceforth are based on physical specimen counts.

Most of the insect inquilines that we identified were mosquito larvae (Culicidae, 772 individuals), followed by midges (Ceratopogonidae, 69 individuals), and a few individuals belonging to the sub-order Brachycera (possibly representatives of the family Phoridae, 13 individuals). All of the culicid larvae in our samples were identified as the genus *Tripteroides*, which we classified into four distinct morphospecies based on characteristics of spines and setae (Supplementary Fig. 2). Mites in the family Histiostomatidae are known *Nepenthes* inquilines [87] and our 18S data revealed their presence (94% of mite OTU sequence counts), but the only mites we could see were in a different lineage: the order Oribatida. This is likely because oribatids are relatively large-bodied and pigmented, while histiostomatids are generally smaller and transparent, and thus more likely to be overlooked or lost when being filtered from the pitcher fluid. We are uncertain whether the oribatid mites function as inquilines or prey, though the latter appears more likely given their lack of obvious aquatic adaptations compared to Histiostomatidae [88]. The prey spectrum is dominated by ants; there were a total of 1172 ants across all samples (mean ± standard deviation = 75.4 ± 35.5) compared to a total of 122 prey items identified as other insects (mean ± standard deviation = 3.70 ± 4.16). We identified 42 morphospecies of ants (3 identifiable to named species) in 5 subfamilies and 15 genera (Supplementary Table 2). The non-ant prey included insects from 11 different orders (Supplementary Table 3). Even after ants were removed, Hymenoptera (primarily Chalcidoidea) was the most frequently encountered order (18 samples), followed by Diptera (13 samples), Coleoptera (12 samples), and Hemiptera (10 samples); the remaining orders each occurred in four or fewer pitchers.

Morphospecies-level richness of culicids, ants, and ordinal richness of non-ant insect prey was not significantly correlated with elevation (Table 4, *p* > 0.05 in all cases). There were no significant effects of the other measured factors on richness (Table 4, *p* > 0.05 for ants and non-ant insect prey), except that culicid morphospecies-level richness increases with decreasing pH (glm, *z* value = − 2.89, *p* = 0.004).

**Table 4:** Results of tests on relationships between insect morphospecies/order-level richness (based on counts of physical specimens) and the other examined factors. All factors were included in a single generalized linear model for each individual arthropod category (ant morphospecies, culicid morphospecies, and prey insect orders not including ants), using a Poisson regression

Factor	Culicid morphospecies		Ant morphospecies		Non-ant prey orders	
	*z* value	*p* value	*z* value	*p* value	*z* value	*p* value
Elevation	− 1.253	0.210	1.234	0.217	0.349	0.727
pH	− 2.890	0.004*	− 1.933	0.053	− 0.878	0.380
Canopy openness	0.030	0.976	− 1.706	0.088	− 1.172	0.241
Fluid volume	2.030	0.042	0.535	0.593	− 0.696	0.486
Pitcher length	− 1.714	0.086	− 1.784	0.074	− 0.235	0.814
Pitcher morph (uppers relative to lowers)	0.401	0.688	0.116	0.908	− 0.550	0.583
Pitcher color (red relative to green)	0.251	0.802	0.188	0.851	− 2.044	0.041

*Significant at a Bonferroni-corrected alpha level of 0.0167

Culicids, mites, and ants were significantly less abundant at high elevations (Table 5, *p* < 0.001 in all cases). Brachyceran larvae and non-ant insect prey exhibit no significant trends in abundance; however, there was a slight increasing trend for the latter (Poisson regression, *z* value = 1.972, *p* = 0.049).

**Table 5:** Results of tests on relationships between arthropod abundance (counts of physical specimens) and all examined factors. All factors were included in a single generalized linear model (along with elevation) for each individual arthropod category (culicids, ceratopogonids, brachyceran larvae, mites, ants, and other insects), using a Poisson regression

Likely Inquilines	Culicids (*n* = 722)		Ceratopogonids (*n* = 69)		Brachyceran larvae (*n* = 13)	
Factor	*z* value	*p* value	*z* value	*p* value	*z* value	*p* value
Elevation	− 5.236	1.65E-07*	− 1.853	0.064	1.419	0.156
pH	− 12.329	2.00E-16*	− 2.057	0.040	− 3.166	0.002*
Canopy openness	10.661	2.00E-16*	3.158	0.002*	− 1.317	0.188
Fluid volume	14.559	2.00E-16*	− 1.421	0.155	0.715	0.475
Pitcher length	− 13.491	2.00E-16*	− 6.373	1.86E-10*	− 0.646	0.518
Pitcher morph (Uppers relative to Lowers)	− 0.356	0.722	2.533	0.011	− 1.206	0.228
Pitcher color (Red relative to Green)	− 0.906	0.365	4.091	4.30E-05*	− 2.015	0.044
Likely prey	Mites (*n* = 87)		Ants (*n* = 1172)		Other insects (*n* = 122)	
Factor	*z* value	*p* value	*z* value	*p* value	*z* value	*p* value
Elevation	− 4.141	3.46E-05*	− 18.349	2.00E-16*	1.972	0.049
pH	− 3.624	2.90E-04*	− 4.244	2.19E-05*	− 4.148	3.36E-05*
Canopy openness	3.284	0.001*	− 16.015	2.00E-16*	− 1.592	0.111
Fluid volume	3.219	0.001*	− 10.619	2.00E-16*	− 0.201	0.841
Pitcher length	− 2.539	0.011	6.928	4.25E-12*	− 1.622	0.105
Pitcher morph (uppers relative to lowers)	−6.109	1.00E-09*	3.278	0.001*	− 0.883	0.377
Pitcher color (red relative to green)	− 0.245	0.806	− 7.363	1.80E-13*	−2.608	0.009

*Significant at a Bonferroni-corrected alpha level of 0.008

Abundances of culicids, brachyceran larvae, ants, other insect prey, and mites all decreased with increasing pH (Table 5; culicids, mites, ants, other insects: *p* < 0.001; brachyceran larvae, *p* = 0.002). The abundances of culicids, ceratopogonids, and mites all significantly increase with canopy openness (Table 5; *p* < 0.001 for culicids and mites, *p* = 0.002 for ceratopogonids), whereas ants decrease with increasing canopy openness (Table 5, *p* < 0.001).

## Discussion

Bacterial and eukaryotic communities inhabiting the same *Nepenthes mindanaoensis* pitchers change with elevation in different ways. Bacterial community composition was much more strongly influenced by pH than by elevation, while elevation had the greatest effect on eukaryotic community composition. Moreover, when numerically dominant metazoan sequence data are removed from the eukaryote OTU table, non-metazoan eukaryotes are still strongly impacted by elevation in our study: the trend is not strictly driven by the macroscopic/multicellular members. This difference between the two domains could be due to the stark physiological differences between them, such as the greater metabolic diversity of bacteria, which grants them wider niche breadth [42].

Elevation and Euclidian distance covary in our study, preventing the separation of elevational effects from potential effects of spatial distance; however, there is evidence suggesting that spatial distance plays little role in pitcher community composition. First, we do not find a distance-decay pattern for either bacteria or eukaryotes, suggesting that neither group is dispersal-limited within our transect [81, 82]. Second, despite greater distances between pitchers at high elevation in the transect, they do not differ in the degree of pitcher-to-pitcher turnover compared to the low elevation pitchers. This demonstrates that community composition is fairly stable within elevation categories, although different between them. The fairly small total geographic distance of our transect (~ 4.2 km) may effectively reduce any effect of physical distance that could potentially shape community structure at larger scales. Nevertheless, we found biologically meaningful patterns that are consistent with effects of elevation found in other studies.

In support of previous findings, we generally see arthropod abundances decreasing with increasing elevation. This has been documented for aquatic insects [91], mites [92], and ants [93]; and corresponds with changes in temperature and precipitation gradients. The notable exception here is that abundance of non-ant insect prey items slightly increases with elevation (Table 5)—possibly a better reflection of the biology of prey capture rather than that of the relative abundances of the insects themselves (Supplementary Discussion). While eukaryotic community composition and the abundances of individual members (whether sequence-based or specimen-based) change with elevation, alpha diversity (i.e., morphospecies richness or effective number of species sensu Jost [70]) does not significantly change with elevation for any of our higher taxa, including bacteria, micro-eukaryotes, and arthropods. Elevation may have a smaller effect on richness in our study system because *Nepenthes* phytotelm communities are comprised of specialized members and have a relatively small potential species pool compared to other environmental DNA samples [65].

A particularly striking change in the eukaryotic taxonomic composition is that Cryptophyceae seems to be replaced by Stramenopiles (primarily Chrysophyceae) at high elevation (Fig. 3a). Past studies of algal communities in high-altitude lakes, using either molecular or morphological methods, found Chrysophyceae to be the most important members of those communities, and Cryptophyceae were relatively less important both in terms of richness and abundance [37, 92–94]. In contrast, Cryptophyceae are more common and diverse elsewhere [18, 95]. Grossman et al. [37] suggested that Chrysophyceae function as an indicator taxon because they reflect the general response of aquatic protist communities to elevational gradients. That phytotelmata such as pitchers can reflect the macroecology of freshwater lakes is intriguing. Tolotti et al. [93] show that Chrysophyceae thrive in high-altitude lakes because they are well-adapted to the oligotrophic conditions common in such lakes. In our study, the abundance of ants, which are the pitchers’ main insect prey, significantly decreases with elevation. It is thus quite possible that the fluid in pitchers at high elevation is less nutrient-rich than low elevation pitchers due to the scarcity of prey in the fluid.

Differences in light requirements could also influence algal patterns [96]. Canopy openness has a significant correlation with elevation in our study, as pitchers grew in more shaded microenvironments at higher elevations. However, canopy openness does not necessarily explain the influence of elevation overall. While some responses are concordant between elevation and canopy (i.e., culicid, ceratopogonid, and mite abundance, Table 5), others are not (i.e., ant abundance, Table 5), possibly reflecting individual ecological differences (Supplementary Discussion).

The plant-regulated trait with the strongest effects in our study was pH. *Nepenthes mindanaoensis* exhibits a wide pH range: samples measured from 6.5 down to the very acidic pH 1.5. This matches previous observations that the *Nepenthes* species with the lowest pH levels also tend to have the greatest pH variance [59]. Fluid pH is the only measured factor with significant effects on bacterial community composition, and it also influences eukaryotic community composition, though to a lesser degree than does elevation. The strong response of bacteria to pH fits with the well-known narrow pH requirements of bacteria [97]. Higher pH levels may require fewer adaptations, thus allowing for greater diversity. This is reflected by the higher 16S-based bacterial alpha diversity at higher pH levels in our study. OTU-based relative abundance analysis verifies this, as the characteristically acidophilic family Acetobacteraceae [98] dominates at low pH, particularly *Acidocella* (Fig. 2), making up as much as 99.8% of bacterial sequences within a given fluid sample.

In contrast with most bacterial groups, but similar to Acetobacteraceae, we found that abundances of all arthropod groups tend to increase with decreasing pH. Dipteran inquilines are likely to be specially adapted to the pitcher digestive fluid environment. For example, many pitcher-associated larvae are specialized [59], including *Tripteroides* where all described *Nepenthes*-associated species have an obligate association [99]. However, it is still somewhat surprising that not only the abundance but also the richness of culicids increases with decreasing pH. This suggests that all four culicid morphospecies are equally tolerant of acidic conditions, rather than the existence of multiple species with different physiological tolerances that only co-occur in more moderate conditions. Prey capture induces fluid acidification [100, 101], and an increase in nutrient availability could explain the relationship between high abundance of ants, other insect prey, and mites with low pH.

For over a century, ecologists have studied elevational patterns of biodiversity in plants and animals, yet only in the past decade have microbes received similar scrutiny. The extent to which microbes abide by the ecological laws of macrobes is still an open question. Few published studies of community changes across an elevational gradient have investigated phytotelmata [102–105], and none of these have compared microbes with macrobes. Aquatic microbial systems have also been understudied in macroecology relative to soil microbes [106], and protists are understudied relative to bacteria [18]. Thus, our work contributes to advancing knowledge of microbial macroecology in multiple ways, and has led to several novel insights. We find that high elevation *Nepenthes* algal communities are analogous to those of alpine lakes. Additionally, we have compared patterns of the living inquiline phytotelm community and their interactions with the external environment and the plant with those of the prey (Supplementary Discussion). Hence, our study provides insight into a wide range of taxa within a small aquatic ecosystem, both living and dead, and how they are affected by external conditions and plant-regulated traits. We can neither confirm nor deny that “everything is everywhere” à la Baas-Becking [7], as neither macrobes nor microbes (whether bacterial or eukaryotic) appear to be dispersal-limited across our transect. However, we can say that “the environment selects,” but the key point is that different taxa experience the same environment differently. For arthropods and eukaryotic microbes (especially algae), the external environment of the elevational gradient is primarily what selects, whereas for bacteria it is the immediate chemical environment within the pitcher that primarily selects. Thus, in multi-taxon macroecology studies moving forward, it will be important to ask not merely “does the environment select?” but also “what is the relevant spatial scale of environmental factors for a given taxon?”

## Electronic Supplementary Material

ESM 1 (PDF 146 kb)

ESM 2 (PDF 169 kb)

ESM 3 (XLSX 11 kb)

ESM 4 (XLSX 17 kb)

ESM 5 (XLSX 10 kb)

ESM 6 (DOCX 20 kb)

## Data Availability

Associated data including spreadsheets, R scripts, and Automontage photographs will be made available on Harvard Dataverse (https://doi.org/10.7910/DVN/JQHLAT). Raw sequence data is available in the NCBI Sequence Read Archive (https://www.ncbi.nlm.nih.gov/sra/PRJNA607326).
