# Supplementary material for: Investigation of an Elevational Gradient Reveals Strong Differences Between Bacterial and Eukaryotic Communities Coinhabiting Nepenthes Phytotelmata
Source: Microb Ecol. 2020 Apr 14;80(2):334–49. doi: 10.1007/s00248-020-01503-y (PMC7371667; doi:10.1007/s00248-020-01503-y)
Supplement: Supplementary file 1 — (PDF 146 kb) [file 248_2020_1503_MOESM1_ESM.pdf]

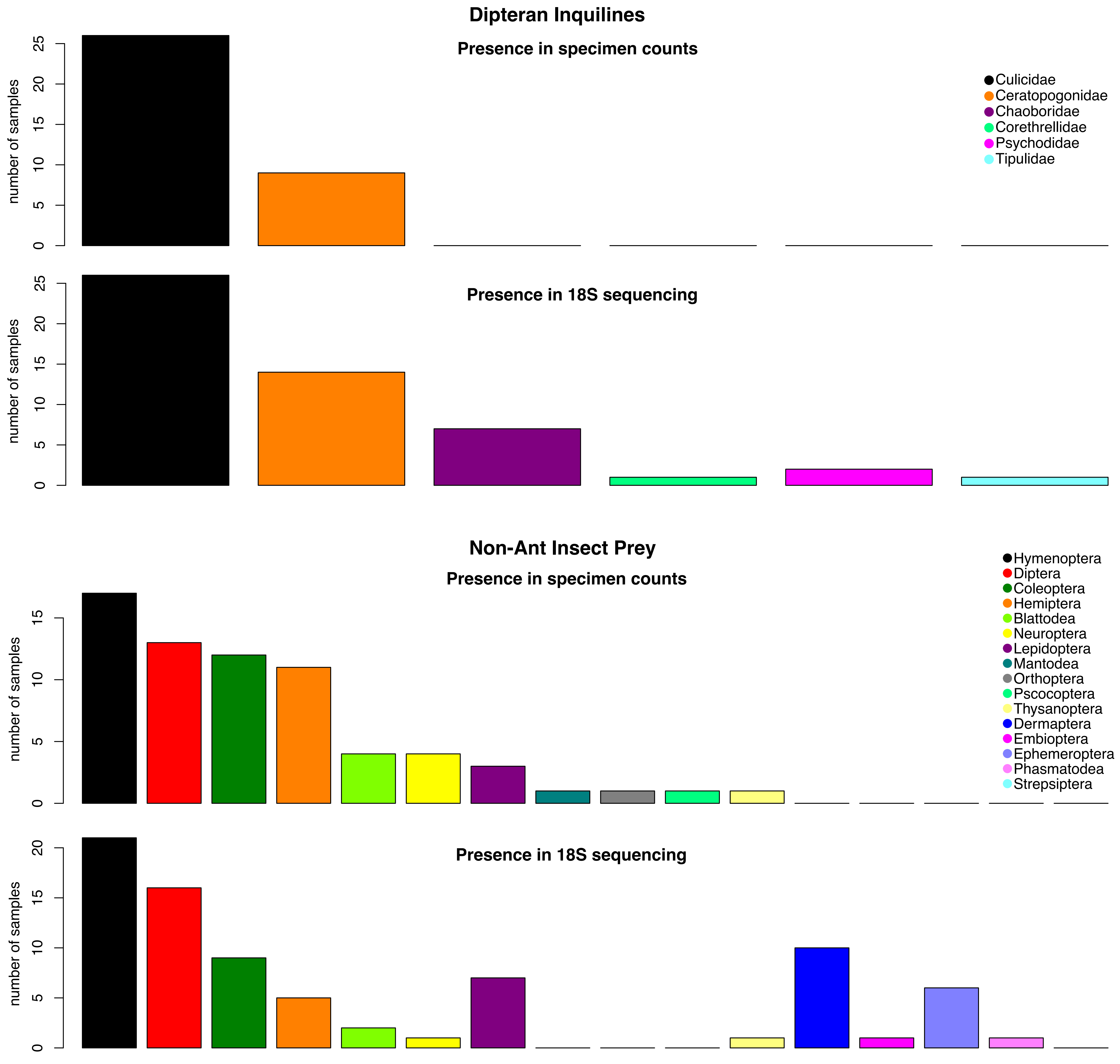
Dipteran Inquilines
Presence in specimen counts
Presence in 18S sequencing
number of samples
Culicidae
Ceratopogonidae
Chaoboridae
Corethrellidae
Psychodidae
Tipulidae
Non-Ant Insect Prey
Hymenoptera
Diptera
Coleoptera
Hemiptera
Blattodea
Neuroptera
Lepidoptera
Mantodea
Orthoptera
Pscocoptera
Thysanoptera
Dermaptera
Embioptera
Ephemeroptera
Phasmatodea
Strepsiptera
