# Supplementary figures and images for: Investigation of an Elevational Gradient Reveals Strong Differences Between Bacterial and Eukaryotic Communities Coinhabiting Nepenthes Phytotelmata
Source: Microb Ecol. 2020 Apr 14;80(2):334–49. doi: 10.1007/s00248-020-01503-y (PMC7371667; doi:10.1007/s00248-020-01503-y)

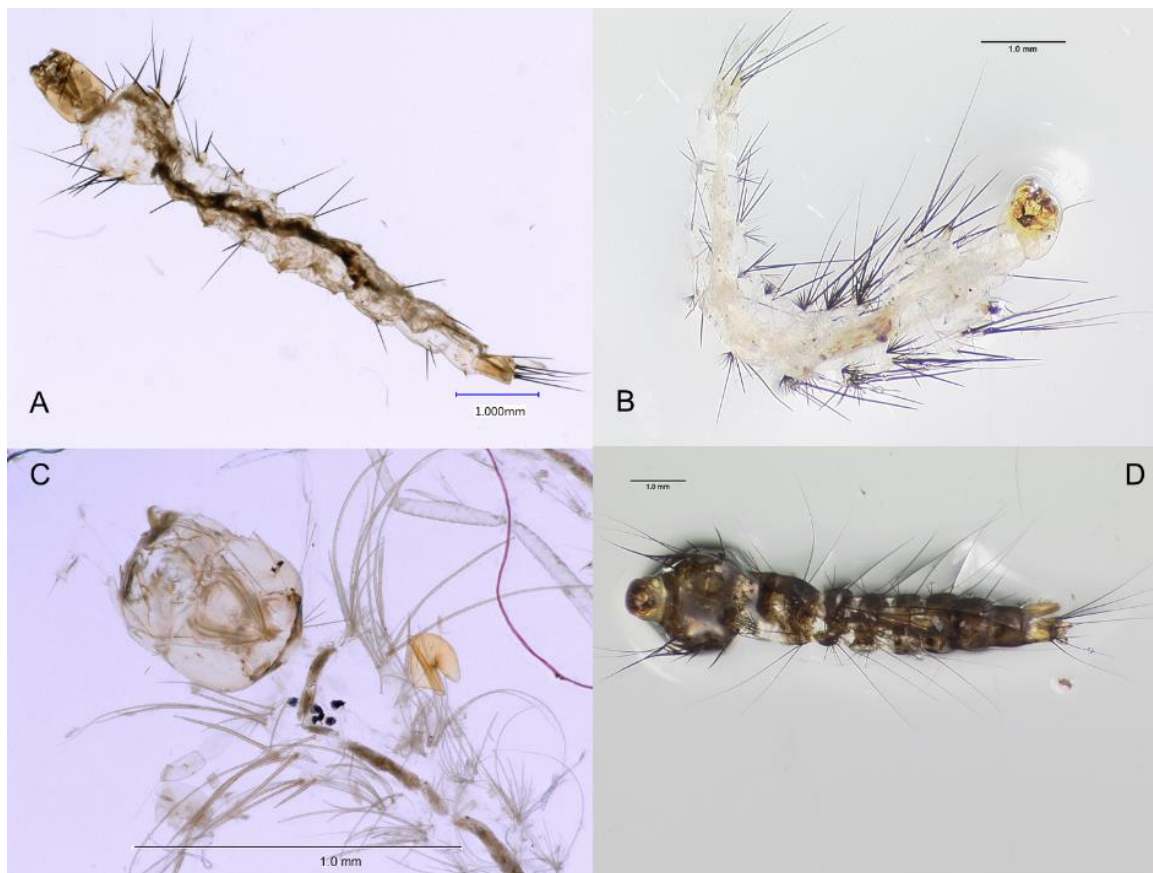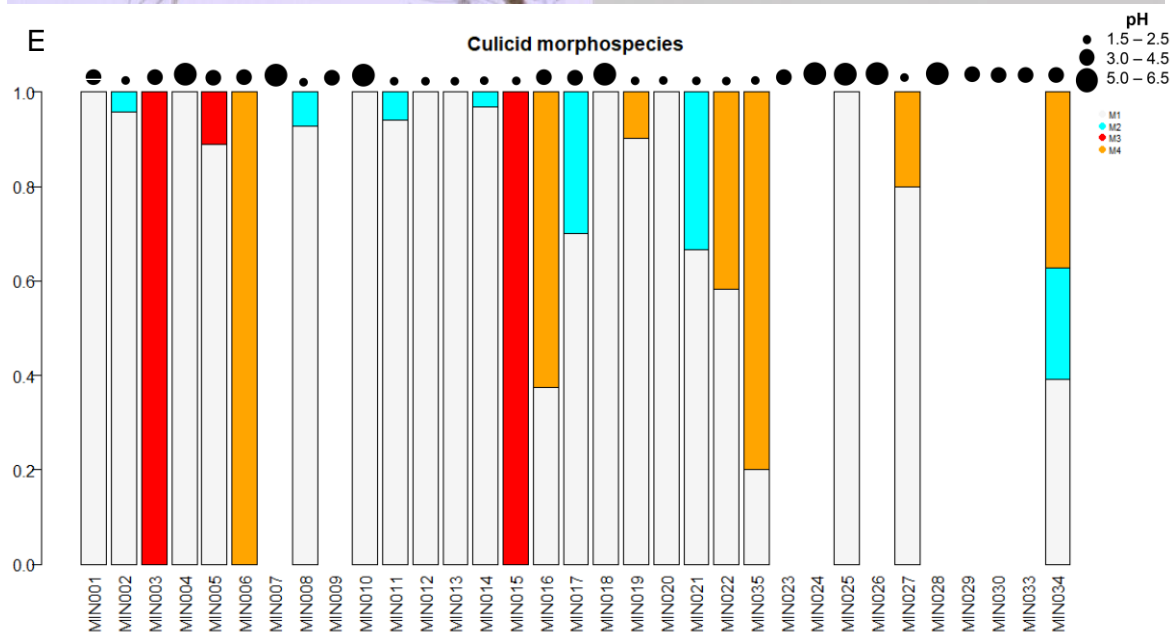

Supplement: Supplementary file 2 — (PDF 169 kb) [file 248_2020_1503_MOESM2_ESM.pdf]
