# Supplementary material for: Investigation of an Elevational Gradient Reveals Strong Differences Between Bacterial and Eukaryotic Communities Coinhabiting Nepenthes Phytotelmata
Source: Microb Ecol. 2020 Apr 14;80(2):334–49. doi: 10.1007/s00248-020-01503-y (PMC7371667; doi:10.1007/s00248-020-01503-y)
Supplement: Supplementary file 6 — (DOCX 20 kb) [file 248_2020_1503_MOESM6_ESM.docx]

**SUPPLEMENTARY DISCUSSION**

We assessed pitcher contents along several axes: comparing the microbes to the macrobes found in these common environments, and also comparing physical counts of inquiline and prey arthropods. Counts of insect prey are useful because pitchers may act as passive pitfall traps that give an accurate picture of diversity patterns for the entire insect fauna in the surrounding landscape. Alternatively, prey composition may not reflect overall patterns of insect diversity if pitchers target prey non-randomly [1]. As dead insect prey are not part of the living community within a pitcher, they are not regulated by the environmental conditions within pitchers in the same way as insect inquilines. Thus, non-interacting biota can in a sense serve as a “control” when examining patterns of inquiline community structure. To our knowledge, the simultaneous analysis of living and dead biota within an ecosystem in a natural context along an environmental gradient is novel, although a recent study of non-native populations of *Sarracenia purpurea* in Europe examined pitcher plant insect prey along an altitudinal gradient [2]. As our study is multifaceted with varied data and analyses, we removed text relating to the prey/inquiline distinction and other side aspects from the main manuscript. Although the following results and discussion points are digressions from the main goals of the study, these are interesting patterns that contribute to an understanding of the *Nepenthes* phytotelm system and raise points for future study, thus we chose to include the following discussion points in this supplement.

**Elevation: Abundance of non-ant insect prey**

In contrast to the other arthropods, non-ant insect prey did not decrease in abundance with elevation, but surprisingly showed a slight increasing trend with elevation (Poisson regression, z value= 1.972, p= 0.049). Rather than acting as neutral pitfall traps that simply reflect the broader patterns of the insect fauna of their surroundings, pitchers can target a particular prey spectrum, as in specialists like the termite-trapping *N. albomarginata* [4]. As ants are often the most abundant arthropods in terrestrial ecosystems [5], many *Nepenthes* species use them as their main source of prey [6]. Whether a diet of ants is achieved solely by a generalist strategy, or whether some species have evolved to specifically target them is an open question [1]. For species like *N. mindanaoensis* that rely mostly on ants, growing at high elevations may necessitate a shift to alternative prey as the abundance of ants can drop off much more steeply than other insects [5, 7]; thus, it would certainly be advantageous for pitchers to increase their ability to capture non-ant prey at higher elevations.

**Canopy openness**

Canopy openness (which includes shading not only by canopy trees, but also understory vegetation covering pitchers as well) decreases with increasing elevation. *N. mindanaoensis* pitchers grew in more shaded light microenvironments at high elevation, including under dense herbaceous foliage and within sheltered nooks and crevices in the ground. Rather than finding sequence-based abundance of photoautotrophic microbes like cyanobacteria and algal eukaryotes being driven by canopy openness, we found that Acetobacteraceae (specifically an *Acidocella* OTU) was significantly more abundant under more open canopy. This is sensible however, as the family contains some known photoheterotrophs [8]; and two of the major algal taxa in our dataset, Chrysophyceae (Stramenopiles) and Cryptophyceae, also include members that are non-photosynthetic [9, 10]. For arthropods, we found that the abundance of culicids, ceratopogonids, and mites all significantly increase with canopy openness, whereas ants decrease with increasing canopy openness. The result for ants makes sense as many of the ant genera we found are at least partly arboreal [11], such as the most abundant genus *Crematogaster* (total count = 420 workers classified into six morphospecies); this is also supported by the significantly higher abundance of ants in upper pitchers.

**Fluid volume and pitcher length**

Phytotelmata act as aquatic “islands” within a terrestrial matrix, as such, one might expect the communities to follow the predictions of island biogeography theory (IBT) [12]. For example, IBT predicts that larger fluid volume (i.e. greater habitat area) would lead to greater species richness or abundance. Past studies have indeed found such an effect of fluid volume in phytotelmata, including for microbes [13]. However, volume in our study had no influence on the community composition of either bacteria or eukaryotes (with or without Metazoa), nor on the morphospecies-/order-level richness of any of the arthropods. However, the specimen count-based abundances of both culicids and mites significantly increase with increasing volume following the expectation based on habitat size. Conversely, ants show the opposite pattern, decreasing with increasing volume. This is difficult to explain other than considering that ants are definite prey, not living inquilines, and thus fluid volume does not represent habitat size in their case. On the other hand, ant abundance increases with increasing pitcher length, a proxy for pitcher size, which shows that bigger pitchers can hold more prey. The aquatic inquilines (culicids and ceratopogonids) decrease with increasing pitcher length, possibly because the depth of the fluid column might influence how well oxygen diffuses to the bottom. Pitcher inquilines may be oxygen sensitive [14], but we lack data on oxygen levels in pitchers and physiological tolerances of *Nepenthes* inquilines in particular.

**Pitcher morph and color**

Pitcher morph and color are two morphological factors that may influence insect inquilines and prey through signaling. In the case of morph, this can encompass both visual and olfactory modalities [15], but also involves the microhabitat differences between the morphs. In this study, we found that ceratopogonid abundance is greater in red pitchers and ant abundance is greater in green pitchers. This suggests a possible signaling function of red pigmentation for ceratopogonid inquilines, and contradicts previous findings of red coloration as a prey attractant [16] in the case of ants, however the many possible roles of red pigmentation in *Nepenthes* are not fully understood [17]. Regarding pitcher morph, mite abundance is greater in lower pitchers and ant abundance is greater in upper pitchers. These results are sensible as the mites counted from our samples are largely oribatids, which are typically soil-dwelling and thus live in closer proximity to the terrestrial lower pitchers. Many of the ants, on the other hand, are at least partly arboreal and thus in closer proximity to the twining upper pitchers. Interestingly, pitcher morph is the one factor that correlates significantly with overall eukaryotic alpha diversity (18S-based), with greater alpha diversity in lower pitchers. This shows that the ecological differences between pitcher morphs can have community-level effects [18, 19]. The one eukaryotic OTU with significant differential abundance by pitcher morph via the ANCOM test is one assigned to *Voromonas* (Protalveolata: Alveolata), which is relatively more abundant in lower pitchers, a trend we found to be generalizable to Alveolata as a whole. Alveolata in our dataset includes gregarines, which are obligate parasites of arthropods [20]; this has ecological implications for the inquilines [21].

**References**

1. Chin L, Chung AY, Clarke C (2014) Interspecific variation in prey capture behavior by co-occurring Nepenthes pitcher plants: evidence for resource partitioning or sampling-scheme artifacts? Plant Signaling & Behavior 9: e27930.

2. Littlefair JE, Zander A, de Sena Costa C, Clare EL (2019) DNA metabarcoding reveals changes in the contents of carnivorous plants along an elevation gradient. Molecular Ecology 28:281–292.

3. Bittleston L (2018) Commensals of *Nepenthes* pitchers. Carnivorous Plants: Physiology, Ecology, and Evolution. Oxford University Press. DOI: https://doi. org/10.1093/oso/9780198779841.003 23:

4. Moran JA, Merbach MA, Livingston NJ, et al. (2001) Termite prey specialization in the pitcher plant *Nepenthes albomarginata*—evidence from stable isotope analysis. Annals of Botany 88:307–311.

5. Hӧlldobler B, Wilson EO (1990) The Ants. Harvard University Press

6. Juniper BE, Robins RJ, Joel DM (1989) The Carnivorous Plants. London, etc.: Academic Press

7. Szewczyk T, McCain CM (2016) A systematic review of global drivers of ant elevational diversity. PLoS One 11: e0155404.

8. Komagata K, Iino T, Yamada Y (2014) The family Acetobacteraceae. The Prokaryotes: Alphaproteobacteria and Betaproteobacteria 3–78.

9. McFadden G, Gilson P, Hill D (1994) *Goniomonas*: rRNA sequences indicate that this phagotrophic flagellate is a close relative of the host component of cryptomonads. European Journal of Phycology 29:29–32.

10. Grujcic V, Nuy JK, Salcher MM, et al. (2018) Cryptophyta as major bacterivores in freshwater summer plankton. The ISME journal 1.

11. General DM, Alpert GD (2012) A synoptic review of the ant genera (Hymenoptera, Formicidae) of the Philippines. Zookeys 1–111. doi: 10.3897/zookeys.200.2447

12. MacArthur RH, Wilson EO (1963) An equilibrium theory of insular zoogeography. Evolution 17:373–387.

13. Bell T, Ager D, Song J-I, et al. (2005) Larger islands house more bacterial taxa. Science 308:1884. doi: 10.1126/science.1111318

14. Bradshaw WE, Creelman RA (1984) Mutualism between the carnivorous purple pitcher plant and its inhabitants. American Midland Naturalist 294–304.

15. Moran JA (1996) Pitcher dimorphism, prey composition and the mechanisms of prey attraction in the pitcher plant *Nepenthes rafflesiana* in Borneo. Journal of Ecology 515–525.

16. Schaefer HM, Ruxton GD (2008) Fatal attraction: carnivorous plants roll out the red carpet to lure insects. Biol Lett 4:153–5. doi: 10.1098/rsbl.2007.0607

17. Gilbert KJ, Nitta JH, Talavera G, Pierce NE (2018) Keeping an eye on coloration: ecological correlates of the evolution of pitcher traits in the genus *Nepenthes* (Caryophyllales). Biological Journal of the Linnean Society 123:321–327.

18. Clarke C (1997) The effects of pitcher dimorphism on the metazoan community of the carnivorous plant *Nepenthes bicalcarata* Hook. f. The Malayan Nature Journal (Malaysia)

19. Ratsirarson J, Silander Jr JA (1996) Structure and dynamics in *Nepenthes madagascariensis* pitcher plant micro-communities. Biotropica 218–227.

20. Chen W (1999) The life cycle of *Ascogregarina taiwanensis* (Apicomplexa: Lecudinidae). Parasitology Today 15:153–156.

21. Baker CC, Bittleston LS, Sanders JG, Pierce NE (2016) Dissecting host-associated communities with DNA barcodes. Phil Trans R Soc B 371:20150328.
